# Signatures of early frailty in the gut microbiota

**DOI:** 10.1186/s13073-016-0262-7

**Published:** 2016-01-29

**Authors:** Matt Jackson, Ian B. Jeffery, Michelle Beaumont, Jordana T. Bell, Andrew G. Clark, Ruth E. Ley, Paul W. O’Toole, Tim D. Spector, Claire J. Steves

**Affiliations:** Department of Twin Research & Genetic Epidemiology, King’s College London, St Thomas’ Hospital Campus, 3rd & 4th Floor South Wing Block D, Westminster Bridge Road, London, SE1 7EH UK; School of Microbiology and Alimentary Pharmabiotic Centre, University College Cork, Cork, Ireland; Department of Molecular Biology and Genetics, Cornell University, Ithaca, NY USA

## Abstract

**Background:**

Frailty is arguably the biggest problem associated with population ageing, and associates with gut microbiome composition in elderly and care-dependent individuals. Here we characterize frailty associations with the gut microbiota in a younger community dwelling population, to identify targets for intervention to encourage healthy ageing.

**Method:**

We analysed 16S rRNA gene sequence data derived from faecal samples obtained from 728 female twins. Frailty was quantified using a frailty index (FI). Mixed effects models were used to identify associations with diversity, operational taxonomic units (OTUs) and taxa. OTU associations were replicated in the Eldermet cohort. Phenotypes were correlated with modules of OTUs collapsed by co-occurrence.

**Results:**

Frailty negatively associated with alpha diversity of the gut microbiota. Models considering a number of covariates identified 637 OTUs associated with FI. Twenty-two OTU associations were significant independent of alpha diversity. Species more abundant with frailty included *Eubacterium dolichum* and *Eggerthella lenta*. A *Faecalibacterium prausnitzii* OTU was less abundant in frailer individuals, and retained significance in discordant twin analysis. Sixty OTU associations were replicated in the Eldermet cohort. OTU co-occurrence modules had mutually exclusive associations between frailty and alpha diversity.

**Conclusions:**

There was a striking negative association between frailty and gut microbiota diversity, underpinned by specific taxonomic associations. Whether these relationships are causal or consequential is unknown. Nevertheless, they represent targets for diagnostic surveillance, or for intervention studies to improve vitality in ageing.

**Electronic supplementary material:**

The online version of this article (doi:10.1186/s13073-016-0262-7) contains supplementary material, which is available to authorized users.

## Background

The ultimate goal of ageing research should be to increase health-span, mitigating a lifespan burdened by morbidity. To this end, frailty is a useful indicator of overall health deficit, describing a physiological loss of reserve capacity and reduced resistance to stressors [1, 2]. It predicts adverse health states such as hospitalisation, dependency and mortality better than chronological age [3], and is increasingly important given the ageing global population with UN estimates of 1.2 billion people aged over 60 years by 2025 [4].

The gut microbiome is the collective coding capacity of the >100 trillion bacteria which significantly enriches the metabolism of the human ‘superorganism’ [5]. It is highly variable between individuals [6], with substantial heritable elements [7], and relatively stable within a healthy adult over time [8]. Inflammation of the gut is associated with disruption of the gut microbiome [9, 10]. Since the gut is the largest interface with external microbes, and frailty is associated with chronic inflammation [11], it is likely that the gut microbiome has a role in frailty.

Age and frailty influence both the composition and function of the gut microbiome in mice [12]. Similarly in humans, significant differences have been observed between the composition of the adult and elderly adult microbiota [13]. When investigating the effects of frailty, significant differences in the abundances of 17 gut microbes were found between 10 highly frail and 13 ‘low frail’ individuals aged over 70 years, from the same care home who shared the same diet [14]. In the larger Eldermet study, the faecal microbiota composition and diversity of 178 older adults varied with level of health dependency. Patients in long-stay continuing care had a less diverse microbiota than short stay, or community dwelling older adults. Dietary intake differed significantly by residence location, and appeared to drive microbiota associations [15]. Across the whole cohort, in various residence locations, the main axis of microbiota composition change correlated with frailty. We recently showed that discrete configurations of microbial taxa can be robustly defined, several of which have distinctive associations with long-term care, frailty and inflammation [16]. These analyses have focused on generally older, more dependent, participants.

Here we aimed to identify associations between frailty and the gut microbiota within a large cohort of younger community dwelling female twins, adjusting for possible confounding effects such as diet, and genetic and environmental factors shared by twins. We describe significant associations between frailty and microbiota diversity and composition, which represent potential diagnostic markers or therapeutic targets for interventions to reduce frailty in ageing.

## Methods

### Frailty index

Frailty was quantified through the Rockwood Frailty Index (FI), which translates meaningfully from an epidemiological perspective to clinical studies [17]. The FI was created as a proportion of deficits [18], using data from the Healthy Ageing Twin Study [19]. Thirty-nine domains of binary health deficit were created from questionnaire data and clinical tests covering a range of aspects of physiological and mental health (Additional file 1: Table S1).

### Microbiota composition

Faecal samples were collected, bacterial DNA extracted, amplified, sequenced and processed as part of a previous study (see methods therein) [7]. Quality filtering and phylogenetic analysis was performed using QIIME 1.7.0 [20]. OTUs were assigned using closed reference clustering with Greengenes v13_5 at 97 % sequence similarity using UCLUST, resulting in the exclusion of 6.2 % of the total sequences that did not cluster to the reference [7].

OTUs that were observed in fewer than 25 % of individuals were not considered for further study. From a total set of 9,840 OTUs (after removing singletons) 16 % passed this threshold, reflective of the sparseness of the data, resulting in a final set of 1,587 OTUs that were used in association analyses. This threshold was applied to focus on associations within abundant OTUs where data sparsity would be less influential on analyses.

### Mixed-effects models

The lme4 package in R was used to generate linear mixed-effects models [21]. The FI was root normalised. Technical covariates included sequencing run and number of sequences in each sample. Biological covariates included relatedness (measured by twin family and zygosity), habitual diet (quantified as the first five PCs from food frequency questionnaires (FFQs) previously assigned to different dietary niches within TwinsUK) [22], alcohol intake, smoking status, age and BMI. Sequencing run and familial traits were modelled as random effects. The Anova function in R was used to compare the ability of models with and without the variable of interest to predict the appropriate response.

Alpha diversity was quantified as observed OTU counts and Shannon and Simpson diversity indices. These were used as response variables to assess associations with alpha diversity.

Models were compared with and without frailty for their ability to predict the log transformed relative abundances of OTUs. Zero counts were handled by addition of an arbitrary value (10^−6^). Modelling was repeated adjusting for alpha diversity to identify associations independent of overall decreases in diversity. The Shannon metric was chosen, as it considers OTU abundance, was not greatly influenced by sequencing depth and was normally distributed. *P* values were adjusted for multiple testing of OTUs, by false discovery rate (FDR) correction using the ‘qvalue’ package in R [23]. This was similarly carried out on the relative abundances of collapsed species and genera.

### Non-parametric correlation

Non-parametric analyses of FI and OTU abundance associations were carried out using the Kendall’s rank sum correlation method in R. The root transformed FI was correlated against the residuals of OTU abundance from previously described mixed models excluding FI. The ‘qvalue’ package was used for FDR correction with significance at 5 %.

### Discordant twin analysis

This was used to demonstrate a lack of bias and potential non-genetic effects. FI discordance between twins was determined where the difference in the pair’s root normalised FI was greater than one standard deviation of the population’s. Paired Wilcoxon signed-rank tests were used to compare the abundance of OTUs between discordant twins. *P* values were Bonferonni adjusted with a significance threshold of *P* <0.05. This was carried out for MZ twins only, DZ twins only and both combined.

### Co-occurrence modules

The R package WGCNA was used to cluster OTUs by co-occurrence [24]. This has been used previously to select OTU modules from pre-calculated co-occurrence matrices [25]. However, in this instance, we also used WGCNA functions to quantify co-occurrence using Pearson correlation between the log transformed relative abundances of OTUs. The pickSoftThreshold function was then used to identify an adjacency threshold (17) above which the edges of the network created a scale-free topology. This adjacency matrix was converted to a signed topological overlap matrix (TOM) using the TOMsimilarity function. Hierarchical clustering of OTUs was performed from a dissimilarity matrix derived from the TOM to generate a dendrogram. Modules were selected using the cuttreeDynamic function to select 24 groups of co-occurring OTUs, containing at least 10 members each. Eigenvectors representing each module were generated using the moduleEigengenes function, to obtain the first PC from the module’s OTU abundance matrix. A dendrogram of module dissimilarity was generated from a Pearson correlation matrix between the eigenvectors, and modules found to be at least 80 % similar were merged. This resulted in 22 final modules, for which eigenvectors were recalculated. The WGCNA cor function was used to calculate Pearson correlation coefficients between these eigenvectors and phenotypic traits.

### Eldermet replication

OTU-level metadata associations were investigated in data from the Eldermet study [15], considering 280 elderly individuals with a mean age of 78 years (age range, 64–102 years). The dataset included community-dwelling individuals, outpatient day-hospital visitors, and short-term and long-term care dwelling individuals.

The Eldermet reference sequences were mapped to the reference sequences from all OTUs, both significant and non-significant, that were assigned to species significantly associated with frailty in TwinsUK. Where there was more than one match between the datasets, the highest scoring BLAST result was retained. Only results above 97 % similarity across the intersect of the sequences were used in subsequent analyses.

Mapped OTUs in the Eldermet dataset were tested for significant association to frailty using a DESeq2 statistical model [26], whereby OTUs were said to be significantly differentially abundant between individuals with a Barthel score <10 compared to a Barthel score of 10 or greater in the Eldermet study if their adjusted *P* value was less than 0.05.

## Results

Frailty, microbiome and complete covariate data were available for 728 women from the TwinsUK cohort. The mean age of the cohort was 63 years (age range, 42–86 years) [7]. The FI followed the expected gamma distribution (Additional file 2: Figure S2) [18], had a mean of 0.116, with 103 pre-frail individuals (FI >0.20) [27]. Thus, on average, members of this cohort have low frailty indices reflective of their age and community dwelling status.

### Frailty negatively associates with gut microbiota diversity

Linear mixed-effects modelling of alpha diversity versus FI adjusting for age, diet, alcohol intake, smoking, BMI and technical covariates, showed frailty had a significant negative association with alpha diversity (Table 1). It was the most influential determinant of alpha diversity for all metrics considered except observed OTU count, which was influenced by the number of sequences (more deeply sampled sequences having more low abundance OTUs).

**Table 1:**
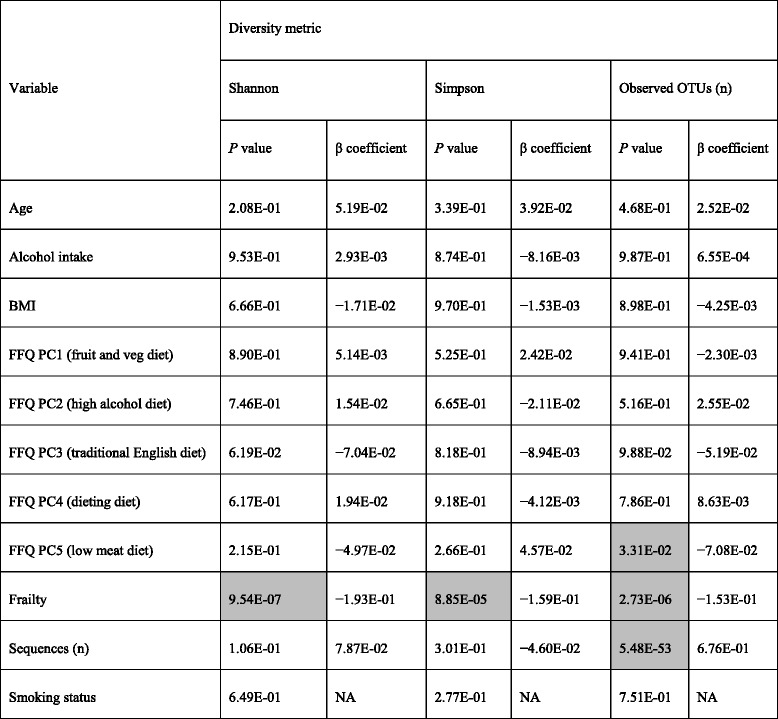
Alpha diversity associations with covariates and the frailty index

Mixed effects models were created with Shannon index, Simpson index or number of observed OTUs as the response. ANOVA was used to compare models with and without each variable. Comparisons where *P* <0.05 are highlighted

### OTU abundances that associate with frailty

Models were extended to investigate frailty effects on OTU abundances. Significant associations were found with 637 OTUs using FDR 5 % (Additional file 3: Table S3). Negative associations were enriched for Clostridiales, in particular Ruminococcaceae, with *Faecalibacterium prausnitzii* the most abundant species level assignment (21 OTUs, all negatively associated). Positively associated OTUs that could be assigned at the species level included *Eubacterium dolichum* and *Eggerthella lenta*.

Modelling was repeated also including Shannon diversity as a covariate. This was carried out to identify frailty specific associations that did not result from the overall decrease in alpha diversity; for instance, cases where a microbe’s abundance is reduced due to reduced abundance of cooperative taxa rather than frailty effects directly. After adjustment for alpha diversity, 22 OTUs remained significant at FDR 5 % (Additional file 3: Table S3). Nineteen belonged to the order Clostridiales; 11 of which were assigned to the family Lachnospiraceae and eight to Ruminococcaceae, although the direction of association was not consistent at the family level. Two OTUs were assigned to the order Erysipelotrichales and one to Coriobacteriales. Three OTUs were assigned species-level taxonomy corresponding to *F. prausnitzii* (FDR q = 0.027 Beta = −0.14), *E. dolichum* (FDR q = 0.013 Beta = 0.17) and *E. lenta* (FDR q = 0.048 Beta = 0.13).

The FI was constructed from measurements taken 0–3 years before faecal sampling. We would expect a monotonic increase in frailty with age [18], so differences in the time between FI quantification and sample collection between individuals should not significantly influence results and only serve to reduce associations. However, to ensure time passage had no effect, OTU level modelling was repeated including the time difference between the faecal and FI measurements as a covariate. All previously identified OTUs retained significance, with the number of significant OTUs increasing slightly to 672.

### Differential OTU abundance between discordant twins

**Fig. 1:**
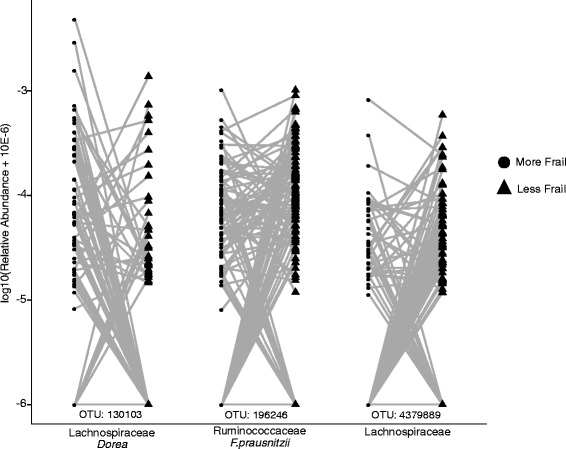
OTU abundances significantly different between twin pairs discordant for frailty. Pairwise plots of abundance between twin pairs discordant for frailty are shown for OTUs that were significant at *P* <0.05 after Bonferonni adjustment considering both MZ and DZ pairs. Paired Wilcoxon rank-signed tests were used to compare abundances for the 22 significantly associated OTUs associated with FI after alpha adjustment. Three were significantly different; the *Dorea* OTU was significantly increased in frailer twins (*P* <10^−4^), whilst the *F. prausnitzii* and Lachnospiraceae OTUs were significantly lower (*P* = 0.001 and *P* <10^−3^, respectively). Note there are overlapping data points where multiple twins had zero counts (log abundance −6) for OTUs

To account for genetics and environmental influences within twins, the difference in abundance of the 22 OTUs significant after alpha diversity adjustment was examined between 111 pairs (65 DZ and 46 MZ) discordant for frailty. Three OTUs showed significantly differential abundance (Fig. 1). An OTU assigned to the genus *Dorea* was the only OTU more abundant in frailer twins (*P* = 4 × 10^−3^). Two OTUs were less abundant in the frailer individuals, which were the *F.prausnitzii* OTU (*P* = 0.03) and one assigned to the family Lachnospiraceae (*P* = 4 × 10^−3^). No OTUs were significantly different considering the discordant MZ subset only and the *Dorea* OTU was the only OTU significant within DZ pairs alone. In cases where an OTU was not detected in one twin within a pair, absence generally followed the expected direction from previously observed associations.

### Frailty associates with species and genus abundance

OTU counts were collapsed by shared taxonomic assignment at the species, genus and family level. The abundances of taxonomic groups were modelled with frailty as a predictor (Additional file 4: Table S4). Twelve species traits were significantly associated with frailty at FDR 5 %. The three most significant were *F. prausnitzii*, *E. dolichum* and *E. lenta*. Other collapsed species were largely from the order Clostridiales and negatively associated with FI. *E. dolichum* and *E. lenta* positively associated with frailty. Only *E. dolichum* remained significant after adjustment for alpha diversity.

**Fig. 2:**
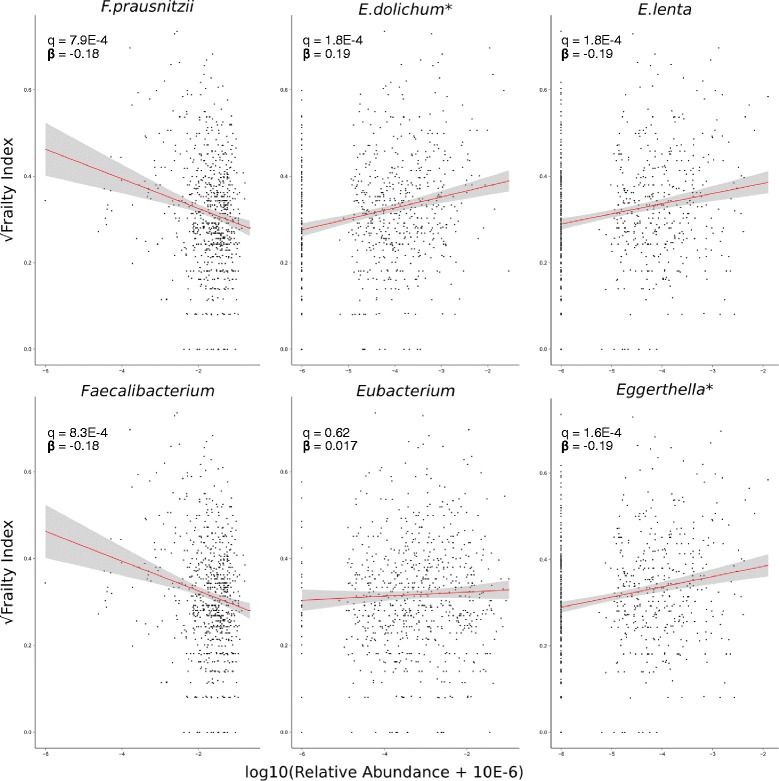
Species abundance associates with FI. Shown are the unadjusted relative abundances versus FI for all species traits that were significantly associated with FI in mixed effects models, and had complete species name assignment. Below, similar plots for their parent genera. *Represents taxa whose association remained FDR significant after adjustment for alpha diversity. Q-values and coefficients shown are without alpha adjustment. Trends are highlighted by linear regression lines shown in red with 95 % confidence intervals shaded in grey. OTUs contained within these taxonomies retained significance within non-parametric analyses

Twelve genera were FDR significant without alpha adjustment. Ten were negatively associated with FI, seven belonging to the order Clostridiales. The only genera positively associated with FI were *Coprobacillus* and *Eggerthella*, the latter the only genus significant after alpha adjustment. The direction of association with frailty of collapsed species and genera containing *F. prausnitzii*, and *E. lenta* reflected those of their constituent OTUs, whereas the *Eubacterium* genus was not significantly associated with FI (Fig. 2). Two collapsed families were significantly associated with FI at FDR 5 %; both belonged to the class Mollicutes but were non-significant after alpha adjustment.

A number of species were not detectable (classified as absent) within the extremes of frailty, for example, *E. dolichum* and *E. lenta* within less frail individuals. We carried out non-parametric analyses to ensure this effect was not inflating significance in mixed models. This was carried out at the OTU level where absence is most prevalent. Associations between OTU abundance and FI were reassessed using Kendall’s rank sum correlations (Additional file 5: Table S5). A total of 138 OTUs were significant at FDR 5 %. All were found in the 637 OTUs identified without alpha adjustment, except seven newly identified OTUs that were all assigned to the family Ruminococcaceae. They also contained 16 of the 22 OTUs significant after alpha adjustment, including the *F. prausnitzii*, *E. dolichum* and *E. lenta* OTUs. This shows that using non-parametric methods, where absence is less influential, the top hits remained significant.

### Replication in the Eldermet cohort

We sought to replicate associations using data from the Eldermet cohort [15, 16], a study that used different methods for sequencing and taxonomic assignment and quantified frailty differently using the Barthel index. Eldermet is also a frailer, more elderly and more dependent cohort, including men and women.

To maximise overlap, OTUs were selected within TwinsUK at the species level. That is, all OTUs that were assigned to species that were significantly associated with FI within TwinsUK were considered for replication regardless of the OTU’s association. Representative sequences from these OTUs within TwinsUK were matched to representatives of OTUs in the Eldermet dataset. Of 638 TwinsUK OTUs considered, 435 mapped to 191 OTUs in the Eldermet cohort. A number of OTUs mapped to the same OTU in the Eldermet set and the remaining 203 had less than the required 97 % similarity to the Eldermet representative sequences and could not be mapped.

Of these 191 mappable OTUs, 96 were significant FI-associated OTUs in TwinsUK. Within the Eldermet cohort, 61 of the 96 were significantly differentially abundant between individuals with a Barthel score of less than 10 compared to a Barthel score of 10 or greater (adjusted *P* value <0.05) as defined the by DEseq statistical methodology. The association to frailty was consistent for all but one of these results.

Within the 60 OTUs that replicated across the two studies, 55 were negatively associated with FI and five positively associated with FI. Of the negatively associated, 54 of the 55 were Clostridia/Clostridiales with the final OTU being Mollicutes/RF39.

The five positive associations were *E. dolichum*, *E. lenta*, two Ruminococcaceae and another Clostridia/Clostridiales. This is consistent with observations in the previously published Eldermet studies that showed populations of co-abundant OTUs that contain a large number of unclassified Clostridiales being both positively and negatively associated with frailty and biological ageing [16].

Of the TwinsUK significant variables that failed to reach significance in the Eldermet cohort, 23 of the 35 had an association to frailty that was consistent in direction between the two studies.

TwinsUK OTUs that belonged to significant species but were non-significantly associated at the OTU level, mapped to 95 OTUs in the Eldermet cohort. Of these, 38 had a significant association to frailty in the Eldermet cohort but the association to frailty was inconsistent, with 11 of the 38 showing the opposite association. This shows that the non-significant TwinsUK OTUs have an inconsistent association in the two datasets and so species level associations are poor markers of frailty.

These results highlight the advantage of carefully identifying a set of high quality predictors of frailty using the twin cohort. The significant OTUs returned were consistent in the direction of the association with frailty with the Eldermet observational study.

### OTU co-occurrence modules have contrasting associations between frailty and diversity

**Fig. 3:**
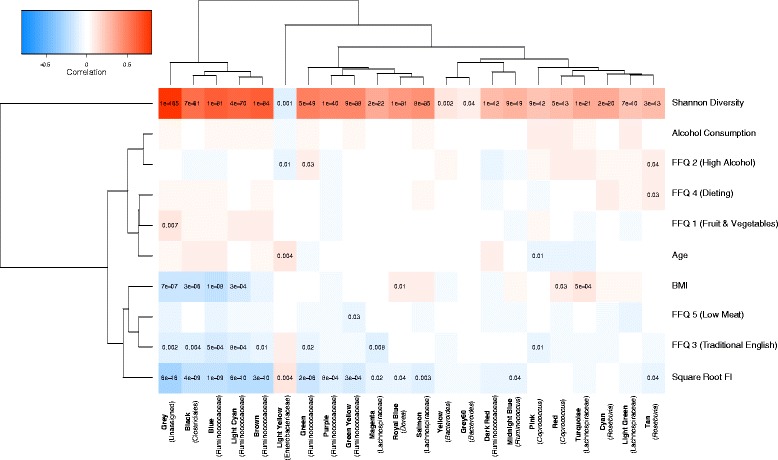
Correlation of frailty, diversity and model covariates with modules of OTUs collapsed by co-occurrence. OTUs collapsed by co-occurrence into 21 modules labelled by colour (grey containing unassigned OTUs). The heatmap displays the correlation between the module eigenvector (ME, representing the combined module abundance as taken from the first PC of the module’s OTUs across all samples) and each variable. Modules and variables are hierarchically clustered using Euclidean distances. Student asymptotic *P* values for significant correlations (*P* <0.05) are shown. The most common taxonomic assignment within a module is shown next to its colour label

Bacteria that share functionality or which are inter-dependent may be taxonomically unrelated but associate similarly with frailty. To overcome this, we collapsed OTUs by co-occurrence, producing 22 co-occurrence modules that were labelled using colour names (the module ‘grey’ contained unallocated OTUs) (Additional file 6: Table S6). Each module represented groups of OTUs with similar abundance profiles across samples, often with similar taxonomic backgrounds. For example, the module coloured brown in Fig. 3 contained 59 Clostridiales OTUs; these included 14 assigned to *F. prausnitzii* which, upon inspection of the representative sequences, were found to share greater than 97 % sequence similarity, highlighting the limitations of OTU clustering and the ability of co-occurrence collapsing to identify similar features.

Eigenvectors representing each module were used to correlate sample traits with module abundance (Fig. 3). Modules showed the strongest associations with FI and alpha diversity, which contrasted in all cases. Modules significantly negatively associated with FI included those coded magenta, royal blue, salmon, midnight blue and tan (Fig. 3); all consisting of OTUs assigned to Lachnospiraceae, in particular that coded royal blue to the genus *Dorea* and tan to the genus *Roseburia*. Other modules negatively associating with FI were black, blue, light cyan, brown, green, purple and green-yellow. All were assigned to Clostridiales OTUs, particularly Ruminococcaceae with brown and green consisting of largely *F. prausnitzii* OTUs. The only module positively associating with frailty was that coded light yellow, containing 13 Enterobacteriaceae OTUs. Clustering traits by their association with modules separated alpha diversity from the other variables. These formed two clusters of differential association, one containing frailty and the other with correlation patterns more similar to those of diversity. BMI, the low meat FFQ PC and the traditional English FFQ PC all clustered with FI. The alpha diversity like cluster contained the remaining dietary PCs, alcohol consumption and age.

## Discussion

Robust associations were identified between frailty and gut microbiota composition. Most notably, FI associated with microbiota diversity. There were also modest associations, both positive and negative, with specific taxa. These findings were robust to adjustment for a range of environmental variables and, in what we believe to be a novel analysis, adjustment for alpha diversity. A number of OTU-metadata associations replicated within the Eldermet cohort.

The distinct negative association between alpha diversity and frailty reflects observations from numerous studies in which predisposition to ill health associates reduced diversity [28–31]. In this study we cannot determine if frailty is the cause, or a consequence of lower microbiota diversity. However, the observed changes in the microbiome could contribute, at least in part, to the detrimental health associated with frailty, particularly in the gut. Should this be the case, maintenance or improvement of gut diversity would be a promising target to encourage health in ageing. This could be achieved through lifestyle interventions to alter factors known to influence diversity, such as diet [32, 33]. This might be particularly effective given the similarity of the observed microbiome associations with diet, BMI and frailty.

We also observed specific taxonomic associations with frailty in the gut microbiota. These were most apparent at the OTU level where a number of significant associations were also observed within the Eldermet cohort, which utilised differing sequencing and analysis platforms, quantified frailty using an alternate index, and used a frailer population [15]. The robustness of our results is supported by their replication within this contrasting data. However, although they were significant, OTU associations were had modest effect sizes. Further studies will be required to investigate the importance of specific taxa in frailty. Our observations provide motivation and direction for such, and are discussed further below.

A number of *F. prausnitzii* OTUs negatively associated with frailty, reflecting previous observations of reduced *F. prausnitzii* abundance in frail or elderly individuals [14, 34]. *F. prausnitzii* is a key butyrate producer [35, 36], thriving in low pH environments created by other short-chain fatty acid producers [37]. There is evidence that *F. prausnitzii* (and butyrate [38]) can have an anti-inflammatory effect on the gut in mice [39, 40]. The observed negative associations of FI with a sub-set of Lachnospiraceae OTUs also support the role of butyrate producers in suppressing frailty-associated inflammation. Some, but not all, Lachnospiraceae genera are butyrate producers [41]; including *Roseburia*, which we identified as negatively correlated with FI when collapsing OTUs by co-occurrence. These observations warrant further investigation into the effects of gut butyrate production on the progression of frailty.

*E. dolichum* and *E. lenta* positively associated with frailty in our experiment. The Eggerthella genus contains a number of pathogenic species, including *E. lenta*, that have been associated with gastrointestinal disease [42–44]. *E. lenta* is also known to harbour a cardiac glycoside reductase operon, which can reduce digoxin, to its more inactive reduced lactone dihydrodigoxin [45].  This drug has been commonly used in frail older individuals to control ventricular rate in atrial fibrillation.  Our finding should stimulate further research as to whether the efficacy and toxicity of cardiac glycosides may be modulated by *E. lenta* abundance.

Turnbaugh and colleagues sequenced the *E. dolichum* genome after observing that its parent class, Mollicutes, was associated with obesity in mice [46]. Its genome was enriched for genes involved in simple sugar processing, which was hypothesised to provide an advantage under a Western diet. The increased abundance of *E. dolichum* are likely a consequence of the significant lifestyle changes, particularly dietary, associated with frailty [15]. However, it is possible that changes to microbiome-encoded metabolism are drivers of frailty. Further studies are warranted to distinguish associations with frailty from those with diet and obesity. Longitudinal observational studies would be of particular use to identify the capacity of specific species to predict an individual’s trajectory of frailty.

While the observed microbiome associations with frailty were robust to discordant twin analyses and replication in an independent cohort, as a cross-sectional study they are not suggestive of cause. To address this, longitudinal frailty and microbial assessments are planned within the TwinsUK cohort. This study was also limited by the determination of the gut microbiota composition using 16S rRNA amplicon analysis, and would be improved through the use of whole metagenome sequencing which provides more accurate species level assignments and direct functional information [5].

This study focused on abundant OTUs to reduce the influence of data sparsity on the analyses. However, by using a closed OTU clustering approach and discarding less abundant OTUs we have not explored the association of rare and unidentified microbiota with frailty. This may be important in future investigations as there may be novel bacterial units specific to the frail state.

We have also not considered the effects of antibiotics and other medication usage on the microbiome, as there was insufficient data available. These effects could potentially confound the observed associations as frailer individuals are more likely to utilize more, and multiple, medications and factors such as antibiotics are known to influence the gut microbiome [47]. This is also being considered in future studies.

The TwinsUK cohort are younger and more able when compared to previously studied cohorts, so these early associations in theory may not apply to frailer individuals. However, replication in the Eldermet cohort, where antibiotic users were excluded, indicates that in part these findings are not restricted to the less frail individuals within TwinsUK. Use of alternate methods in the quantification of the microbiota and frailty within the replication cohort also suggests that observed associations are independent and robust to the methods used.

## Conclusions

We have identified a number of associations between host frailty and the gut microbiota, including modest associations with specific taxonomic abundances and a striking negative association with microbiota diversity. Although more work is required to delineate the direction of effect between frailty and the composition of the gut microbiota, we believe that the associations we have described here provide motivation and direction for such studies. They also provide microbial targets for future investigation, with the ultimate goal to develop the capability to rationally modulate the gut microbiome to improve health in ageing people.

### Ethics

Ethical approval for the HATS (Healthy Ageing Twin Study), from which the frailty data were obtained, and microbiota studies within TwinsUK were provided by the NRES Committee London – Westminster. The Cork Clinical Research Ethics Committee provided approval for the Eldermet study.

### Data availability

Sequence data for samples within this study are available as part of previously published data under the European Bioinformatics Institute (EBI) accession numbers ERP006339 and ERP006342 [7]. Other data are available for request from TwinsUK.

## Additional files

Additional file 1:
**Table S1 of domains used in the construction of the frailty index.** (DOCX 11 kb) Additional file 2:
**Figure S2 showing the distribution of the FI in TwinsUK.** The distribution of the frailty index in the TwinsUK cohort fits the expected gamma distribution. Histogram of untransformed FI values for the 728 individuals included in the study with a fitted gamma distribution shown in red. (PDF 5 kb) Additional file 3:
**Table S3 of significant OTU-FI associations.** (XLS 88 kb) Additional file 4:
**Table S4 of significant taxonomic associations with FI.** (XLS 11 kb) Additional file 5:
**Table S5 of OTU associations with FI in non-parametric analyses.** (XLS 176 kb) Additional file 6:
**Table S6 of OTU module assignments.** (XLS 131 kb)
