# Erratum to: signatures of early frailty in the gut microbiota

**DOI:** 10.1186/s13073-016-0275-2

**Published:** 2016-02-17

**Authors:** Matthew A. Jackson, Ian B. Jeffery, Michelle Beaumont, Jordana T. Bell, Andrew G. Clark, Ruth E. Ley, Paul W. O’Toole, Tim D. Spector, Claire J. Steves

**Affiliations:** 1 Department of Twin Research & Genetic Epidemiology, St Thomas’ Hospital Campus, King’s College London, 3rd & 4th Floor South Wing Block D, Westminster Bridge Road, London, SE1 7EH UK; 2 School of Microbiology and Alimentary Pharmabiotic Centre, University College Cork, Cork, Ireland; 3 Department of Molecular Biology and Genetics, Cornell University, Ithaca, NY USA

## Erratum

It has come to our attention that there is an error in one of the author names for this article [1]. The name of the first author was incorrectly listed as Matt Jackson but the correct name is Matthew A. Jackson. The original article has now been updated and the publisher apologises for any inconvenience caused.
