# Supplementary material for: Signatures of early frailty in the gut microbiota
Source: Genome Med. 2016 Jan 29;8:8. doi: 10.1186/s13073-016-0262-7 (PMC4731918; doi:10.1186/s13073-016-0262-7)
Supplement: Additional file 1: — Table S1 of domains used in the construction of the frailty index. (DOCX 11 kb) [file 13073_2016_262_MOESM1_ESM.docx]

**Additional file 1. Domains incorporated into the Frailty Index**

| **Deficit category** | **Separate domains** |
| --- | --- |
| Co-morbidity | 10 |
| Physical measures | 6 |
| Biochemistry | 5 |
| Mental health | 3 |
| Self-reported general health | 4 |
| Disability | 8 |
| Social functioning | 1 |
| Polypharmacy | 1 |
| Pain | 1 |
