# Supplementary figures and images for: Signatures of early frailty in the gut microbiota
Source: Genome Med. 2016 Jan 29;8:8. doi: 10.1186/s13073-016-0262-7 (PMC4731918; doi:10.1186/s13073-016-0262-7)

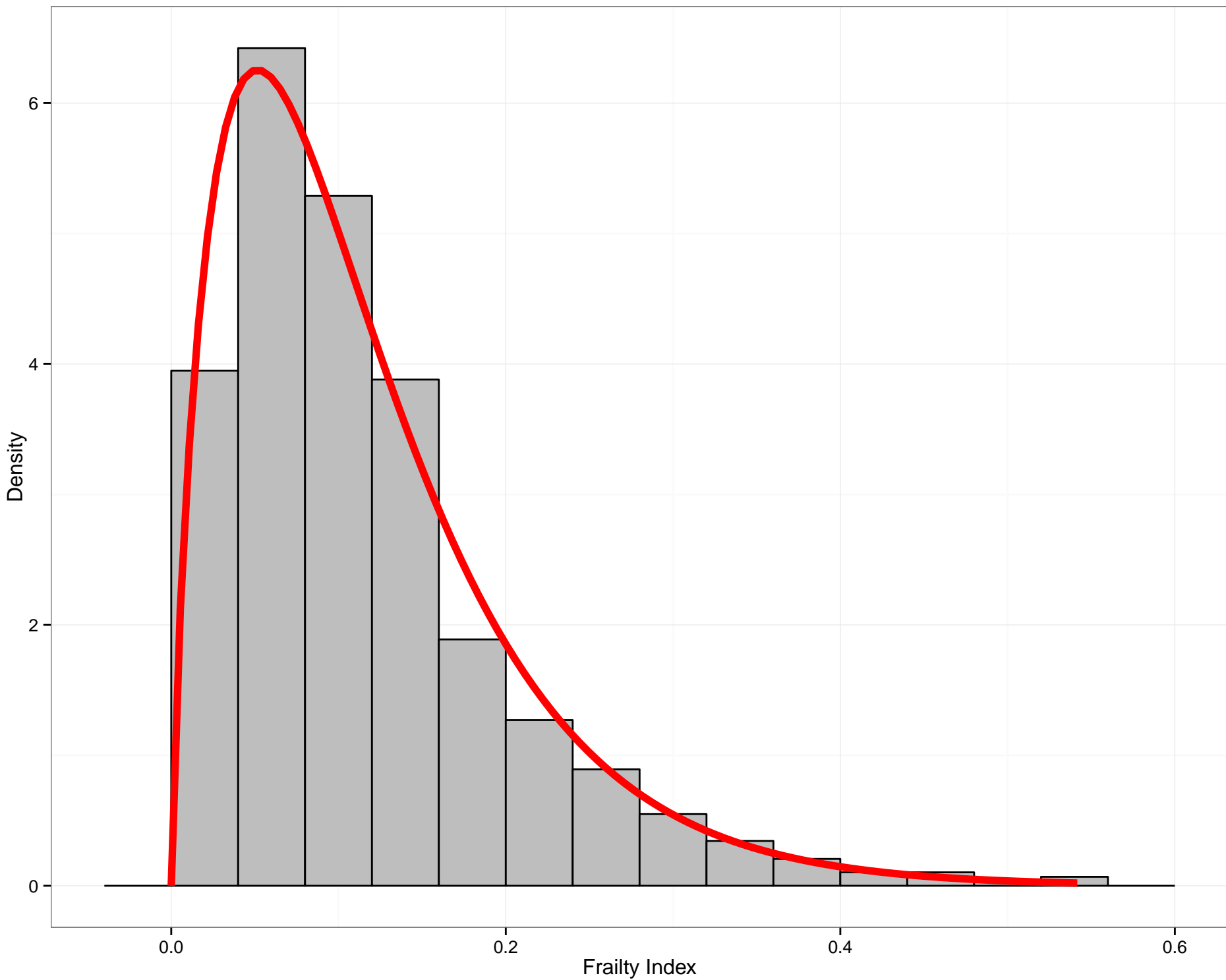

Supplement: Additional file 2: — Figure S2 showing the distribution of the FI in TwinsUK. The distribution of the frailty index in the TwinsUK cohort fits the expected gamma distribution. Histogram of untransformed FI values for the 728 individuals included in the study with a fitted gamma distribution shown in red. (PDF 5 kb) [file 13073_2016_262_MOESM2_ESM.pdf]
